# Spatial Organization Plasticity as an Adaptive Driver of Surface Microbial Communities

**DOI:** 10.3389/fmicb.2017.01364

**Published:** 2017-07-20

**Authors:** Arnaud Bridier, Jean-Christophe Piard, Caroline Pandin, Simon Labarthe, Florence Dubois-Brissonnet, Romain Briandet

**Affiliations:** ^1^Antibiotics, Biocides, Residues and Resistance Unit, Fougères Laboratory, ANSES Fougères, France; ^2^Micalis Institute, INRA, AgroParisTech, Université Paris-Saclay Jouy-en-Josas, France; ^3^MaIAGE, INRA, Université Paris-Saclay Jouy-en-Josas, France

**Keywords:** microbial biofilm, spatial dynamic, structure/function, adaptative response

## Abstract

Biofilms are dynamic habitats which constantly evolve in response to environmental fluctuations and thereby constitute remarkable survival strategies for microorganisms. The modulation of biofilm functional properties is largely governed by the active remodeling of their three-dimensional structure and involves an arsenal of microbial self-produced components and interconnected mechanisms. The production of matrix components, the spatial reorganization of ecological interactions, the generation of physiological heterogeneity, the regulation of motility, the production of actives enzymes are for instance some of the processes enabling such spatial organization plasticity. In this contribution, we discussed the foundations of architectural plasticity as an adaptive driver of biofilms through the review of the different microbial strategies involved. Moreover, the possibility to harness such characteristics to sculpt biofilm structure as an attractive approach to control their functional properties, whether beneficial or deleterious, is also discussed.

## Introduction

The traditional perception of microbes as unicellular life forms has deeply changed over the last decades with the collection of scientific evidences showing that microorganisms predominantly live in dense and complex communities known as biofilms. Biofilms are classically defined as aggregates of cells adhering to a surface or interface and often embedded in an extracellular matrix of polymeric substances. They constitute one of the most successful mode of life on Earth (Flemming et al., 2016). They are consequently found in natural, industrial, medical, household environments and, from the human point of view, they can be either beneficial or detrimental. Indeed, microbial biofilms are involved in essential nutrient cycling or biotechnological processes as well as in severe chronic infections and biodeterioration phenomenon (for instance Beech and Sunner, 2004; Bjarnsholt, 2013; Berlanga and Guerrero, 2016). Positive or negative impacts directly result from the ability of microorganisms to express specific functions in these complex communities compared to the single planktonic state. The higher resistance of biofilm cells to antimicrobials compared to that of their planktonic counterparts is a telling example of such specific functional properties and should be relied to the structural characteristics of the community (Bridier et al., 2011). Indeed, both the microbial growth and the production of matrix lead to the rise of a biological edifice offering progressively a protective structure to inhabitants able to hinder penetration and action of antimicrobials. The development of three-dimensional biofilm structure also generates physicochemical gradients and physiological heterogeneity with slow growth resistant phenotypes for instance (Stewart and Franklin, 2008). Recently, Berleman et al. (2016) demonstrated the central role of multicellular bacterial community structure in the colonization of surface by *Myxococcus xanthus*. Indeed, the authors showed that extracellular polymeric substances (EPS) synthesis led to the creation of microchannels which govern both bacterial motility and cell-to-cell interactions and finally organize multicellular behavior during swarm migration. In contrast, a mutant lacking EPS showed a deficiency of cell orientation and poor colony migration. As biofilms are mostly complex associations of strains and/or species in our environments, spatial arrangement of genotypes within biofilms also governs strain interactions and the evolution of social phenotypes as immediate neighbors in the structure are more affected by the social behaviors (Nadell et al., 2016). Spatial organization of genotypes and social interactions will thus govern the whole community architecture and functions (Liu et al., 2016). Functional properties of a biofilm therefore emerge from the construction and shaping of the microbial structure like many of the emergent properties of natural communities relying on the creation of biogenic structures by habitat-forming organisms (Flemming et al., 2016).

The close relationships between the architecture of a biofilm and its functional properties emphasizes the need to better describe and understand cell behavior, from single cell to multicellular scale, during biofilm structure development and maturation. Recent technological advances in methodologies including imaging and microscopy, molecular techniques, and physico-chemical assays, enabled the development of novel approaches dedicated to biofilm studies (Azeredo et al., 2017). The possibility to observe biofilm using high resolution and non-destructive methods now allows investigating the dynamics of multicellular structure development and the fate of each of its individual cellular components in parallel. For instance, the key architectural transitions and associated biophysical and genetic mechanisms supporting the developmental program of *Vibrio cholerae* biofilms have been recently disclosed using single-cell live imaging (Drescher et al., 2016; Yan et al., 2016). This kind of observations has clearly improved our understanding of spatio-temporal development of biofilms and has finally increasingly supported the intimate connection between structural modulations and the emergence of functional features and survival strategies. Indeed, the ability of biofilms to adapt their structure in response to internal or external stimuli, called hereafter the architectural plasticity, appears as a key factor affecting the fitness of individuals within the whole microbial community. Interestingly, the role of plasticity in bacterial survival was already demonstrated at the cellular scale. Bacteria are able to alter their morphology and to produce specific morphotypes conferring survival advantages in hostile environments. This was showed for numbers of bacterial pathogens for which filamentation is essential in the resistance to phagocytosis and overall for persistence during infection (Justice et al., 2008; Justice et al., 2014).

In this review, we will discuss the central role of architectural plasticity in the emergence of functional properties of biofilms and as a communal bacterial response to many harsh conditions and external attacks. We will also deal with the various mechanisms developed by microorganisms to build and modify the three-dimensional community and, with the existing strategies for humans to sculpt biofilm architecture in order to control their function.

## Biofilm Architecture Plasticity as a Collective Response to Environmental Fluctuations

The starting point of the development of the three-dimensional biofilm structure corresponds to the transition from planktonic state to sessile mode of life which occurs in response to diverse environmental cues and cell-to-cell signaling molecules. The translation of perceived signals to specific genetic expression and finally to a series of dramatic metabolic and phenotypic changes involves complex regulatory networks and diverse molecules including the second messenger cyclic-di-GMP (c-di-GMP) in number of bacterial species (Kostakioti et al., 2013; Romling et al., 2013). A correlation between high intracellular levels of c-di-GMP and biofilm formation has indeed been shown for a variety of species and various biofilm determinants including flagella rotation, exopolysaccharide production, surface adhesin expression, secondary metabolite production, antimicrobial resistance and other stress responses (Valentini and Filloux, 2016). In addition, the quorum sensing (QS), which is a cell-to-cell signaling system making bacteria able to communicate with each other via the production and detection of signaling molecules, enable the regulation of communal behaviors (Srivastava and Waters, 2012). The interconnection between QS and c-di-GMP pathways enables bacteria to act collectively through coordinated response to cellular signals or environmental conditions. It was showed for instance in *V. Cholerae* that QS and c-di-GMP pathways are strongly intertwined at many levels and that their integration play a key role in the control of the expression of *vpsT*, a transcriptional activator that induces biofilms formation (Srivastava et al., 2011). Similarly, Ueda and Wood (2009) demonstrated in *Pseudomonas aeruginosa* that the transcription of the *tpbA* gene encoding a tyrosine phosphatase involved in synthesis of polysaccharides and biofilm formation, is under the direct control of QS while this enzyme is also involved in the regulation of intracellular c-di-GMP concentrations. Such observation clearly highlights the convergence of the two signaling processes and the connection between the environment, cell populations and finally biofilm formation.

Using this sensor system, bacteria are able to coordinate their activities during the different steps of biofilm development leading to complex three-dimensional structures. Recurrent developmental stages can be schematically defined in bacteria ranging from initial adhesion to irreversible attachment, formation of microcolonies, macrocolonies development and maturation of architecture and then dispersion (Monds and O’Toole, 2009). Nevertheless, the development program and its dynamic are actually very specific and largely depend on nutrient conditions, pH, temperature, hydrodynamics conditions, species involved, etc… Fundamentally, the shaping of specific biofilm architecture reflects the impact of local growth conditions (Toyofuku et al., 2015). Numerous studies in various bacterial species reported the impact of temperature, hydrodynamics or nutrient concentration on biofilm structure suggesting an adaptation of biofilm shaping to optimally fit to growth conditions (Stoodley et al., 1998; Yang et al., 2006; Abdallah et al., 2015). The changes of biofilm structure alter the diffusibility of substances and enables metabolic adaptation under various conditions by optimizing nutrient and waste product exchange for instance (Toyofuku et al., 2015).

**FIGURE 1:**
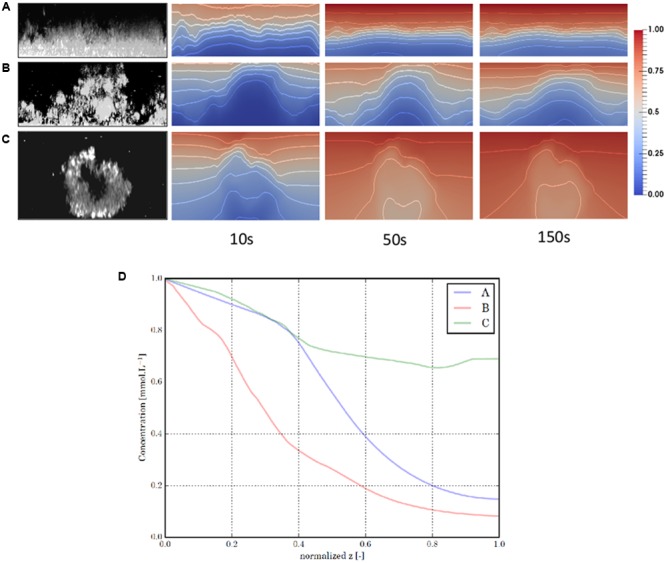
Modeling of diffusion in biofilm of various architecture. CLSM sections of three characteristic biofilm structures were displayed in the first column: **(A)** a flat (*Escherichia coli*), **(B)** a mushroom-like (*Pseudomonas putida and Pseudomonas aeruginosa*) and **(C)** a egg-like structure (*Salmonella enterica*). Those images are used as an input of a modeling pipeline which simulates the diffusion of a chemical component through the biofilm, from a bulk source located in the upper boundary of the image. Based on the biofilm images, we construct for each structure a heterogeneous diffusion coefficient map that reflects the diffusive capabilities of the biofilm: the higher the local bacterial density, the lower the local diffusion coefficient. Next, this tensor is inserted in a reaction-diffusion equation together with a reaction function that mimics the consumption of the component by the bacteria. The consumption rate also varies with the local bacterial density. We display three snapshots of the simulated component distribution, at time *t* = 10, 50, and 150 s when the steady-state is reached. Isolines are displayed every 0.1 to better represent the distribution gradients. We finally display a cut in z of the component distribution in each biofilm at steady-state **(D)**. The cut plane of a given biofilm crosses its point of minimal component concentration at steady-state. To facilitate the comparison, we normalized the z-coordinates of the different graphs. We can see that the biofilm structure is a determinant driver of the component density map at steady-state, presenting a diversity of situation, from quasi-uniform distributions (structure C) to strong gradients (structures A and B).

This is illustrated in **Figure 1** where confocal images of biofilms with various architectures were used as an input of a modeling pipeline, which simulates diffusion of a chemical molecule through biofilm and thus reflects its diffusive capabilities. The diffusion coefficient maps obtained suggested that biofilm architecture is a determinant driver of the chemical compound density map at steady state, presenting a diversity of situations, from quasi-uniform distributions to strong gradients.

Accordingly, it is clear that structural adjustments of biofilm clearly lead to both the modulation of phenotypic heterogeneity and the way each bacterium perceive its local microenvironment. This architectural plasticity provides thereby an efficient way to adapt to various stresses for microorganisms. Many demonstrations of this phenomenon occur in our environments as for instance, stream biofilms in rivers, which dynamically adapt and evolve in response to the streambed environment and flow intermittency through modifications of their physical structure, species composition and through spatial re-organization (Battin et al., 2016; Sabater et al., 2016). The intimate relation between architectural differentiation and community composition suggests that this micro-scale process is an important driver of the biofilm adaptation to the fluctuations of stream conditions, especially to compensate hydrodynamic perturbations and changes in quantity and quality of nutrients (Besemer et al., 2009).

Another concrete illustration of adaptation through biofilm structure modulation is the stimulation of biofilm production in different bacterial species exposed to antimicrobials, metals and a large range of other molecules (Hoffman et al., 2005; Perrin et al., 2009; Shemesh et al., 2010; Marchal et al., 2011; Chen et al., 2015). In many cases, the presence of subinhibitory concentration of such toxic molecules induces the sur-expression of genes coding for matrix components that finally lead to an increase of biofilm production and a modification of its three-dimensional structure (Shemesh et al., 2010). In line with this, it was showed in *Thiomonas* sp. that arsenic exposure lead to an increase of EPS production and cell death within microcolonies creating hollow voids structure that is subsequently followed by active dispersal of cells (Marchal et al., 2011). Authors suggested that the survival and persistence of *Thiomonas* sp. under selective pressure of arsenic exposure relied on its ability to rapidly develop biofilm followed by the dispersal of a more resistant population.

Architectural plasticity of biofilms thus gives the opportunity to bacteria to constantly reorganize their direct microenvironments to face adverse conditions and to better harness surrounding resources (**Table 1**). Structural adaptations can occur through various active processes which mostly involve a differential expression of genes or a genetic plasticity in response to conditions changes. The diverse mechanisms, directly or indirectly involved in the shaping of biofilm architecture, are discussed in the next section.

**Table 1:** Examples of biofilm structural responses to environmental fluctuations associated with the alteration of community functions.

Biofilm composition	Environmental fluctuation	Structure alteration	Impact on functional properties	Reference
*Bacillus subtilis* 3610	Exposition to sublethal dose of chlorine dioxide (ClO_2_)	Increased matrix production and acceleration of biofilm formation	Partial protection against ClO_2_	Shemesh et al., 2010
*Bacillus subtilis* 3610	Exposition to bacilli relatives isolated from soil	Increase in matrix-producing cannibals subpopulation, matrix induction	Hypothetical increase survival within a multispecies biofilm	Shank et al., 2011
*Thiomonas* sp. CB2	Exposition to subinhibitory dose of arsenite	Increased production of extracellular polysaccharides and creation of hollow voids containing motile cells	Increased protection to As(III)	Marchal et al., 2011
*Pseudomonas fluorescen*s PCL1701	Exposition to calcium ions (CaCl_2_)	Increase biofilm surface coverage, biovolume	Reduced stiffness, higher viscous effect, larger adhesive values at the surface of the biofilm	Safari et al., 2014
Stream biofilms	Exposition to flow intermittency	Changes of physical structure, community composition and spatial arrangement	Adaptation of ecosystem metabolism	Battin et al., 2016; Sabater et al., 2016
Gravity sewer biofilms	Increasing shear stress	Increase porosity of the biofilm	Reduction in the chemical oxygen demand	Xu et al., 2017
*Xanthomonas axonopodis* (citrus bacterial canker)	Exposition to *Bacillus subtilis* or *Bacillus* TKS1-1 *amyloliquefaciens WG6-14*	Alteration of the spatial repartition and density of the pathogen in the multispecies biofilm	Citrus leaves protection from the plant pathogen	Huang et al., 2012
*Burkholderia cenocepacia*	Exposition to the free-living ciliate *Tetrahymena pyriformis*	Increase of biofilm production and formation of specific round-shape microcolonies	Resistance to protozoan grazing	Kaminskaya et al., 2007
Fouling biofilm developed on ultrafiltration membrane	Exposition to the protozoa *Tetrahymena pyriformis*	Shift in biofilm structure from flat to aerial and porous 3D organization	Permeate fluxes in the presence of the predators increased by 2	Derlon et al., 2012
Fouling biofilm developed on filtration membrane	Exposition to metazoan worms (nematodes or oligochaetes)	Shift in biofilm structure from flat to aerial and porous 3D organization	Increase of permeate fluxes in the presence of the predators	Klein et al., 2016
*Staphylococcus aureus* RN4220	Exposition to bacilli swimmers	Vascularisation of the biofilm matrix	Sensitization to biocide action	Houry et al., 2012
*Streptococcus pyogenes* SP5	Exposition to fluoroquinolone derivatives	Modulation of EPS production and biofilm architecture	Sensitization to the antibiotic treatment	Shafreen et al., 2011
*Staphylococcus epidermidis*	Exposition to Dispersin B (beta-*N*-acetylglucosaminidase)	Hydrolyze of the glycosidic linkage of the extrapolysaccharidic matrix, biofilm dispersion	Potentialisation of antibiotic (cefamandole nafate) action	Donelli et al., 2007
Water system multispecies biofilm	Exposition to sodium nitroprusside (NO donor)	Drastic reduction in 3D organization	Partial loss of chlorine tolerance	Barraud et al., 2009
*Listeria monocytogenes*	Exposition to DNase I and proteinase K	Disruption of the biofilm matrix, loss of 3D organization	Decrease of persistence on industrial surfaces	Nguyen and Burrows, 2014
*Pseudomonas aeruginosa*	Exposition to biosynthetic glycoside hydrolases PelAh and PslGh	Disruption of the biofilm spatial organization	Potentialisation of colistin and neutrophils	Baker et al., 2016
Wound biofilms*, Staphylococcus epidermidis*	Exposition to EDTA (Ethylenediaminetetraacetic acid)	Disruption of biofilm structure	Potentialisation of antimicrobials	Finnegan and Percival, 2015; Maisetta et al., 2016

## Microbial Systems to Shape Biofilm Structure

**FIGURE 2:**
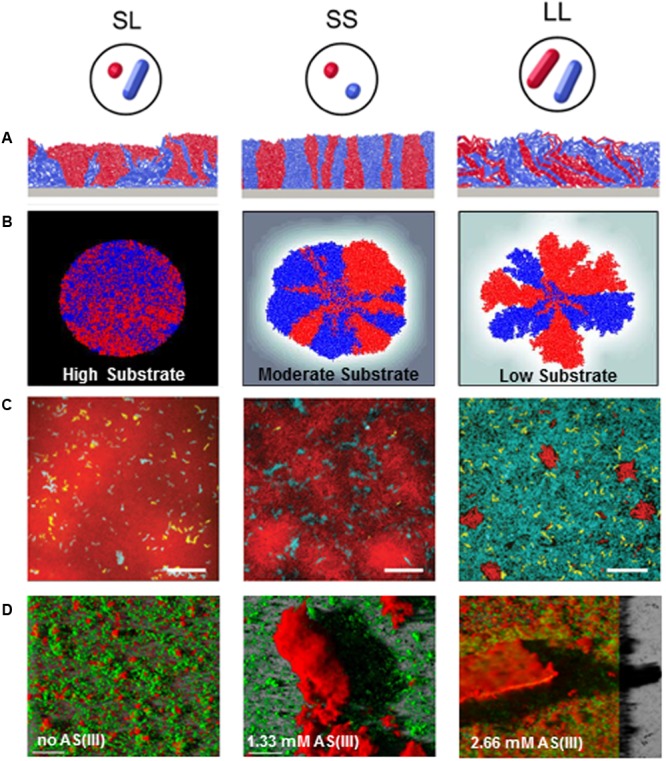
Biofilm architectural modulations in response to environmental stimuli or depending on bacterial composition. **(A)** Impact of cell morphology on biofilm spatial organization. Images displayed 2D sections from simulations where biofilms exponentially grown from 1:1 mixtures of red- and blue-labeled strains form distinct 3D patterns depending on the coccal (S) or rod-like (L) morphology of the strain (Adapted from Smith et al., 2017). **(B)** Impact of substrate availability (High, moderate and low availability) on biofilm architecture and lineage segregation. Simulations were started with a 1:1 mixture of red and blue cells, where cell color served a neutral marker for lineage segregation. Substrate concentration decrease was associated to a higher spatial segregation of cell lineages (Adapted from Nadell et al., 2010). **(C)** Impact of disturbance frequency on *Vibrio cholerae* biofilm spatial organization and strain competition. Images are optical sections taken from the bottom cell layer of biofilms initiated with a 1:1:1 mixture of wild-type strain (teal): a mutant strain hyper-secreting biofilm matrix (red): a mutant strain that is unable to produce extracellular matrix (yellow) cells in microfluidic devices (scale bar: 20 μm). Biofilms grew under continuous nutrient provision (left), or underwent disturbance events every 12 h (middle) or every 6 h (right). Each disturbance event consist in stopping the flow during 2 h to lead to nutrient limitation. Cells were then allowed to disperse to a new microfluidic chamber by pumping the dispersed cells from the initial chamber to the new chamber. After a 2 h-incubation, flow was resumed to pump fresh medium in the newly colonized chamber and enable biofilm growth (adapted from Yan et al., 2017). **(D)** Impact of toxic on EPS production and biofilm structure in *Thiomonas* sp. CB2. Images show three dimensional confocal reconstruction of 7 day-old biofilms cultivated in the absence, or in the presence of 1.33 and 2.67 mM Arsenic [As(III)]. Biofilms were stained using SYTO9 (cells, green) and ConA (exopolysaccharides, red) (Adapted from Marchal et al., 2011).

Microorganisms harness an arsenal of complementary mechanisms to tailor biofilm architecture. They range from regulation of cell motility to modification of cellular morphology, production of matrix components, generation of genetic and physiological heterogeneity or subpopulation interactions. Examples of modulations of biofilm architecture in response of various environmental conditions and depending on bacterial composition are displayed in **Figure 2**.

### Genetic and Physiological Adaptation at Single Cell Scale

Physical and chemical microenvironments within the biofilm (e.g., varied conditions of pH, osmotic strength, nutrients or exposure to sublethal concentrations of biocide) induce heterogeneous metabolic activity and adaptive responses among biofilm cells (Bridier et al., 2011; Giaouris et al., 2015). During biofilm development, the population displays multiple phenotypes (Sauer et al., 2002). At the single cell scale, the diversity of cell properties are due to either the phenotypic adaptation driven by up- or down- regulation of gene expression, or the appearance of genetic mutants driven by an increased level of mutation in biofilm environment.

Gene regulation at different stages of biofilm formation, compared to the free-living mode of life, can be studied through the comparison of transcriptomic (Waite et al., 2006; Moreno-Paz et al., 2010; Guilhen et al., 2016), proteomic (Sauer, 2003; Resch et al., 2006; Vilain and Brozel, 2006; Giaouris et al., 2013; Qayyum et al., 2016) or metabolomic (Wong et al., 2015; Stipetic et al., 2016) profiles revealing up- or down- regulated functions. For example, in mature biofilms of *P. aeruginosa*, more than 50% of proteins are upregulated and more than 100 proteins are *de novo* synthesized in comparison to planktonic cells (Sauer et al., 2002). The multiple phenotypes described in biofilm communities do not correspond to a simple mixture of planktonic cells at different growth stages. The biofilm proteome of *Bacillus cereus* was for example demonstrated as unique and different from those of exponential and stationary-phase planktonic cells (Vilain and Brozel, 2006). Compared lipidomics between planktonic and biofilm cells also support the idea of specific biofilm phenotypes. Indeed, in various growth conditions, the biofilm cell membrane of different bacterial strains was shown to be more saturated than their planktonic counterparts, whatever their growth phase (Dubois-Brissonnet et al., 2016). In addition, the spatialized environments in biofilms promote the generation and fixation of a phenotypic diversity compared to selection of only one or very few clones in well mixed environment (Traverse et al., 2013; Martin et al., 2016). The spatial distribution of the biofilm multiple phenotypes can be visualized within the biofilm thickness through the observation of different patterns of physiological characteristics (growth rate, mRNA, proteins synthesis or CsgD production) using for example Gfp reporter systems (Werner et al., 2004; Lenz et al., 2008; Stewart and Franklin, 2008; Serra et al., 2013).

Regulation of genes which differentiate planktonic and biofilm protein patterns are numerous and can be partitioned in several categories: metabolism (carbon catabolism, aerobic/anaerobic metabolism, membrane and transport), stress responses and adaptation, motility and attachment (flagellin, surface proteins), EPS production and quorum-sensing signaling (Whiteley et al., 2001; Sauer et al., 2002; Khemiri et al., 2016). Both transcriptional and post-transcriptional regulation occur: the first is slow but may be important for the long-term stability of the biofilm (Guttenplan and Kearns, 2013); the second is described to be mainly controlled by the c-di-GMP intracellular level. As mentioned previously, elevated intracellular levels of c-di-GMP generally promote EPS synthesis and biofilm formation, while decreased levels reduce biofilm formation (Martinez and Vadyvaloo, 2014).

Besides, biofilms can constitute an optimal environment for both cell to cell exchanges of genetic material and genetic mutations in biofilm inhabitants. They offer a panel of ideal characteristics for horizontal gene transfer through conjugation and transformation. These include the presence of high cell density favoring physical contact between biofilm bacteria and of a matrix that is rich in communication signals and in extracellular DNA (eDNA) (Madsen et al., 2012). In addition to providing transferable genetic elements (Hannan et al., 2010), the eDNA plays a central role in triggering natural competence in biofilm bacteria (Molin and Tolker-Nielsen, 2003). Horizontal gene transfer has therefore been described in several studies revealing that conjugation levels were 700–1000-fold higher in biofilms compared to planktonic bacterial cells (Król et al., 2013; Savage et al., 2013). This “permeability” of biofilm bacteria to heterologous mobile genetic elements is likely to shape the evolution of biofilm bacteria and to enhance their relatedness (Madsen et al., 2012). Another mechanism yielding genetic evolution in biofilms is linked to a higher mutation rate of certain biofilm bacteria. Important studies down this line have been performed in *P. aeruginosa*. Initial observations reported that genetic diversification occurred through a *recA*-dependant mechanism within short-term growth in biofilms and yielded mutants with multiple novel traits including motility, nutrition requirements, morphology, biofilm phenotypes, and stress resistance (Boles et al., 2004). The study of mutations *in-situ* within biofilms using a *gfp* gene containing a +1 frameshift mutation showed that mutations occurred in microcolony structures and increased at a frequency 100 to 1800-fold higher than that observed in planktonic cultures (Conibear et al., 2009). The underlying mechanism is linked to the mismatch repair system (MRS) which monitors the fidelity of DNA replication and recombination through its two main components MutS and MutL (Oliver et al., 2002). Using *mut*S deficient derivatives of *P. aeruginosa* and a flow-cell biofilm model system, Luján et al. (2011) showed that the mutants yielded enhanced phenotypic and morphological diversities over wild type strains in structured biofilms. Interestingly, the generated morphotypic variants showed increased competitiveness over the parental strain. This is to correlate to the high prevalence (30–60%) of mutator strains due to alterations in the *mut*S and *mut*L genes in *P. aeruginosa* chronic infections while detection of mutators is rare in *P. aeruginosa* acute infections (Gutiérrez et al., 2004; Feliziani et al., 2010).

Altogether, this overall genetic plasticity of bacteria in biofilm yields a rapid development of diversity among members of biofilm communities and is likely to shape the biofilm structure because of the co-development of the different morphologies and phenotypes. This provides the biofilm with what has been termed the “insurance hypothesis” in ecology that considers that the stability of many biological communities relies on their diversity which increases the chance that some members will be able to withstand environmental variations that the community may encounter (Boles et al., 2004). This enhanced clonal diversity in biofilms is a real challenge in the control of pathogen and detrimental biofilms as they may rapidly adapt to environmental stresses such as treatments with antimicrobials (Macià et al., 2011; Koch et al., 2014; Van Meervenne et al., 2014). In contrast, this diversity is a real benefit in biotechnological issues in which biofilms can be exploited in numerous applications and under many different environmental conditions (Berlanga and Guerrero, 2016; Piard and Briandet, 2016).

#### Cell Adaptation with Direct Impact on Biofilm Structure

Individual adaptative responses of biofilm cells, due to heterogeneous environments within their complex living place, lead to individual phenotypic changes, such as individual cell morphology and motility or modification of matrix production.

Bacterial motility, within or outside the biofilm structure, is a major driver of the community plasticity. Once associated to a surface, most of the bacterial cells transfer from a motile to a non-motile state. *P. aeruginosa* for example becomes non-motile as soon as it attaches irreversibly to a surface and forms clusters with non-motile cells during the maturation of the biofilm (Sauer et al., 2002). In accordance, transcriptional profiles of *P. aeruginosa* biofilms showed that motility genes are downregulated compared to planktonic cells (Whiteley et al., 2001). The *B. subtilis* motility is also inhibited under biofilm conditions (Guttenplan et al., 2010). In the short-term, motility is inhibited at multiple levels through accumulation of intracellular c-di-GMP (Ahmad et al., 2013; Guttenplan and Kearns, 2013). In the longer term, regulation relies on transcriptional repression which is slow but may be important for the long-term stability of the biofilm (Guttenplan and Kearns, 2013). In mature biofilms, maintenance of motility for the majority of the cells can destabilize multicellular aggregates and regulation of biofilm plasticity likely shifts to other determinants including EPS production (Guttenplan and Kearns, 2013). Nevertheless, some motile minor isogenic subpopulations can coexist with sessile biofilm cells, creating transients pores within a mature biofilm structure, altering the diffusion-limitation properties of the matrix (Houry et al., 2012; Turonova et al., 2015). In *Campylobacter* biofilms, an unusual continued expression of the motility complex was described by proteomics in the whole population which suggests a crucial role of the measured motility in this biofilm phenotype (Kalmokoff et al., 2006). Similarly, flagellar hook protein (FlgE) was expressed in biofilm cells but not in planktonic cells of *Cronobacter sakazakii* (Ye et al., 2016).

Flagella synthesis and movement are highly regulated in response to environmental conditions. During biofilm maturation, starvation stress occurs in the growing biofilm structure, along with a lack of oxygen and accumulation of by-products and QS signaling molecules. All these factors are important drivers of microbial dispersion (Huynh et al., 2012; Martinez and Vadyvaloo, 2014; Solano et al., 2014). Non-coding small RNAs were also recently identified as players of this dissemination process (Chambers and Sauer, 2013). In the well described *P. aeruginosa* biofilm cycle, dispersion is a consequence of the return to a motile state of a subpopulation of bacterial cells in the center of a cluster. This return is possible through phage-mediated localized cell death (hollow-voids) along with the synthesis of enzymes that can degrade extracellular substances (Webb et al., 2003; Sauer et al., 2004). Dispersion is heterogeneously distributed at the surface of the biofilm and can induce modification of the whole biofilm topography. Motility up- and down- regulation is thus an important driver of the biofilm structure plasticity through its role in attachment, cluster formation and disruption.

Besides, individual cell morphology can also have a great impact on the organization of the population within the biofilm consortium (Smith et al., 2017). Growing in biofilm state, some coccoid-shaped bacteria or small rod can elongate and multiple morphotypes of isogenic cells can appear in different layers. Two different shapes of *Lactococcus lactis* were observed in 16 h flow-cell biofilms: coccoid cells were localized in the depth of the structure while a stratum of elongated filaments rises on the interfacial layers of the structure (Perez-Nunez et al., 2011). Similarly, different morphotypes of uropathogenic *Escherichia coli* were observed from coccoid form to elongated rods, through different stages of biofilm formation. Filamentous bacteria were observed on the edge of late biofilm in connection with detaching cells (Justice et al., 2004). The filamentation was shown to be a response to stressful environment and is essential for uropathogenic *E. coli* virulence (Justice et al., 2006, 2008).

In addition, EPS are the cement of biofilm architecture and their modulation trigger direct alteration of the spatial structure (Ziemba et al., 2016). EPS content includes water and biopolymers originating from biofilm microorganisms including polysaccharides, proteins, lipids, and eDNA (Flemming, 2011; Fong and Yildiz, 2015; Limoli et al., 2015). From an anthropomorphic biofilm perspective, the matrix has been described as the house of bacteria and as such its structure and composition are unique according to the inhabiting bacteria and the environment (Stoodley et al., 1999; Watnick and Kolter, 2000; Flemming et al., 2007; Flemming, 2011). The EPS matrix cannot be considered as a homogeneous slimy material, but rather as the sum of multiple microhabitats with different local environments (oxygen concentrations, pH-values, redox potential, shear forces, etc.). This stratification governs biofilm heterogeneity in which bacterial groups distribute themselves according to their preferred particular microenvironment and to symbiotic relationships (Watnick and Kolter, 2000; Stewart and Franklin, 2008; Flemming, 2011). This heterogeneity in space is doubled by heterogeneity in time: EPS evolves with the biofilm aging and appears as a dynamic structure due to various events including degradation of matrix elements by bacterial enzymes, dissolution of EPS components, incorporation of new material, etc… (Sutherland, 2001). It can also be noted that most of the different components of the matrix are associated by non-convalent interactions suggesting that dissociation can occur through local modifications of the EPS physicochemical properties (pH, ionic strength, hydration, etc…) (Neu and Lawrence, 2016). This poorly characterized plasticity of the EPS matrix makes it the least understood component of biofilms biology and as such has been termed the “dark matter” of biofilms (Flemming and Wingender, 2010; Flemming et al., 2016).

In an attempts to characterize the signals governing matrix formation and modification in biofilms, Shemesh et al. (2010) showed that exposure of *B. subtilis* and *P. aeruginosa* to sublethal doses of a biocide (chlorine dioxide, CIO_2_) stimulate biofilm formation. The transcription of two major operons involved in matrix production [*epsA-epsO* involved in polysaccharide (PS) production and *yqxM-sipW-tasA* involved in amyloid production] was shown to be increased by CIO_2_ via the membrane-bound kinase KinC. Interestingly, *kinC* mutants unable to make a matrix were hypersensitive to CIO_2_. Another kinase within the *epsA-epsO* operon, the EpsAB tyrosine kinase, is involved in regulation of PS production by a seemingly QS mechanism (Elsholz et al., 2014). The membrane sensor EpsA is able to sense the presence of PS and control kinase activity. In the absence of PS, the kinase is inactivated by autophosphorylation while the presence of PS inhibits autophosphorylation and stimulates the phosphorylation of glycosyltransferases and thereby the synthesis of PS. This positive feedback loop therefore ties PS synthesis to the external concentration of PS. This opens exciting perspectives in applications in which exogenous polysaccharides could be used either as inducers of the biofilm way of life or as modulators of the matrix structure. Also this raises the question whether PS produced by one biofilm bacteria could trigger PS production in another biofilm bacterium. A part of the answer probably relies on the yet unknown specificity of the sensor EpsA toward the different PS produced by a biofilm bacterial community. In an attempt to explore such interbacterial interactions, Shank et al. (2011) investigated whether soil bacteria were able to affect the biofilm development in *B. subtilis*. Using a fluorescent reporter fused to the *tapA* promoter, the coculture screening test showed that most strains able to induce matrix production in *B. subtilis* belonged to the *Bacillus* genus suggesting that interactions occur mostly with close relatives. Two mechanisms were dissected. One involves the activation of the sensor kinase KinD while the other is kinase independent and involves the master regulator Spo0A (Shank et al., 2011).

Species belonging to *Thiomonas* species are frequent in arsenic polluted sites and play key roles in arsenic natural remediation (Marchal et al., 2011). Exposure of *Thiomonas* sp. to sublethal arsenite concentration yielded biofilms with an up to six-fold increase in PS production concomitantly to a 83-fold increase in cell death and cell lysis. This was accompanied with a complex rearrangement of the biofilm structure into PS covered mushroom-like structures in which eDNA was a key player as treatment with a nuclease abolished such phenomenon. eDNA is indeed a crucial component of the biofilm matrix and is involved in multiple interactions with other EPS components including PS and amyloids (Hu et al., 2012; Liao et al., 2014; Schwartz et al., 2016). In *Staphylococcus aureus*, the *cidA* and *lrgA* genes act as holins and antiholins, respectively, and regulate cell lysis in an analogous way to that observed in bacteriophage-mediated cell lysis. While wild-type *S. aureus* produced biofilm with distinct mushroom-like 3D structures that are characteristic of mature biofilms, both a *cidA* mutant deficient in lysis and a *lrg* mutant deficient in the inhibition of CidA-mediated lysis produced biofilms lacking 3D mushroom-like structures (Mann et al., 2009). *S. aureus* is also able to produce and secrete Nuc, a thermostable nuclease. Analysis of the biofilm formed by a *nuc* mutant showed increased amounts of mushroom-like structures. Also, treatment of the *S. aureus* biofilms with DNAseI in flow cell chambers completely removed biofilms. Altogether this suggests that different bacterial factors are able to modulate the level of available eDNA that appear critical in the shaping of biofilm structure and dispersal.

#### Cell Adaptation with Indirect Changes on Biofilm Structure via Increased Resistance and Persistence

Physiological adaptation of individual cells within the biofilm community may lead to an increased resistance to biocides and antibiotics. Stresses such as starvation (oxygen or nutrients) in the depth of the biofilm or contact with sublethal concentrations of antimicrobials during disinfection can induce a bacterial stress response and higher tolerance to biocides (Mangalappalli-Illathu et al., 2008; Bridier et al., 2011). The higher individual cell resistance can be explained by several mechanisms. An overexpression of enzymes that are able to degrade biocides (catalase, superoxide dismutase) was described in biofilm under the control of QS (Hassett et al., 1999). *P. aeruginosa* membrane efflux pumps were shown to be up-regulated for cell cultivated in biofilm although their exact role in the biology of these sessile communities needs to be clarified (Zhang and Mah, 2008; Soto, 2013). Moreover, by limiting biocide intracellular penetration, the observed increase in membrane saturation in biofilm cells compared with their planktonic counterparts can be another resistance mechanism (Dubois-Brissonnet et al., 2016). After repeated antimicrobial treatments, the development of the most resistant surviving cells in the biofilm structure will modify the spatio-temporal dimension of the biofilm architecture.

### Interactions between Biofilm Subpopulations

Multispecies biofilm is a result of cell–cell and cell–environment interactions such as cooperation, competition or exploitation that create heterogeneity in biofilms (Liu et al., 2016). These specific interactions between species are involved in the spatial organization of biofilms in which they are more favored than in planktonic environments. They maintain their diversity and stability by generating more physiological and functional heterogeneity (Nadell et al., 2009; Pamp et al., 2009; Rendueles and Ghigo, 2015; Kragh et al., 2016; Liu et al., 2016; Pande et al., 2016). Indeed, in specific environment, some species cannot form biofilm alone, but grow in association with others species in multispecies biofilm showing interspecific cooperation interactions between subpopulations (Palmer et al., 2001). Specific interactions and spatial organization within biofilm create fitness effect through social phenotypes. A telling example is the symbiotic two-species consortium formed by *Pseudomonas putida* and *Acinetobacter* sp. strain C6 which has evolved in a non-random spatial organization where *P. putida* exclusively attached and grew on pre-existent colonies formed by *Acinetobacter* sp. strain C6. Resulting evolved communities were characterized by an increased fitness and productivity (Hansen et al., 2007). Microscopic time-lapse observations revealed that cell clusters were arranged according to a uniform pattern and that such structure results from the moving along the surface and the fusion of early microcolonies (Haagensen et al., 2015). These observations illustrate the improvement of community fitness through the active spatial structuration of its individuals and theirs interactions, and thereby the stabilization of their symbiotic relations.

Similar observations were made by describing the evolution of communities derived from a clonal *Burkholderia cenocepacia* biofilms (Poltak and Cooper, 2011). The authors highlighted the emergence of three variants and their persistence in mixed communities displaying enhanced productivity than any monoculture. The authors demonstrated that such productivity gains were due to the asymmetrical cross-feeding between the different ecotypes and the expansion and restructuration of biofilm space that constructed new niches. Overall, the fitness of cooperative or competitive phenotypes largely depends on neighboring cells that finally influences the spatial arrangement of genotypes within biofilms (Nadell et al., 2016; Stubbendieck et al., 2016). Reciprocally, the spatial structuring of genotypes within biofilm greatly influences the evolution of social phenotypes (Nadell et al., 2016). Many social phenotypes are regulated by QS through the secretion of diffusible signaling peptide (Nadell et al., 2016; Perchat et al., 2016). Studies showed how interspecies QS may have a role in competition interactions. In a *P. putida*–*P. aeruginosa* mixed-species biofilm, it was demonstrated a spatial repulsion between the two isolates (Fernandez-Piñar et al., 2011; Bridier et al., 2014). Indeed, both populations secreted molecules which negatively alter the growth of each other; *P. aeruginosa* secreted quinolone, a QS signaling molecule which inhibits biofilm formation of *P. putida*, and in the same way, *P. putida* secreted putisolvin which is regulated by QS and inhibits *P. aeruginosa* biofilm formation (Diggle et al., 2003; Kuiper et al., 2004; Fernandez-Piñar et al., 2011; Bridier et al., 2014). Other systems can have an important role in interspecies interactions such as communication and transport including outer membrane vesicles (OMVs) (Wang et al., 2015). OMVs could promote bacterial interactions and thereby participate to the architectural integrity of biofilms (Schwechheimer and Kuehn, 2015). In *Helicobacter pylori, Franciscella, P. aeruginosa, V. cholera* and *P. putida*, vesicles are involved in biofilm formation by increasing hydrophobicity of cells surface and by participating to the matrix formation (Yonezawa et al., 2011; Baumgarten et al., 2012; van Hoek, 2013; Altindis et al., 2014; Murphy et al., 2014; Wang et al., 2015). OMVs can also have an interspecies interference property in biofilms when they are coupled with an antimicrobial action and alter bacteria in biofilms (Schooling and Beveridge, 2006). Species interactions contribute thus through various way to shape biofilm architecture. Actually, processes related to intra-species interactions, as for instance cell death, can also play a key role in biofilm structuring. Localized cell death is known to trigger wrinkle formation of biofilm by focusing mechanical forces and instigate vertical extending of the biofilm (Asally et al., 2012; Rendueles et al., 2014; Nadell et al., 2016). Overall, it has been showed that cell death plays an important role in the development of multicellular biofilms and the subsequent dispersal of surviving cells (Webb et al., 2003; Mai-Prochnow et al., 2004). In *Bacillus subtilis* biofilms, subpopulations of cells use a cannibalistic strategy involving the production and secretion of two toxins to lyse sensitive siblings which then provide nutrients for the cannibals. Interestingly, cannibal cells correspond to the subpopulation producing the extracellular matrix, the production of toxins and matrix being triggered by surfactin, a paracrine signal whose production is controlled by the QS signaling peptide ComX (Lopez et al., 2009). This process finally promotes matrix producer subpopulations and enables the development of biofilm structure through an increase of matrix production.

This extraordinary large diversity of means provides to microorganisms the ability to dynamically shape biofilm architecture and functions using complementary mechanisms. Numbers of processes involved in architecture plasticity are thus inter-related through complex regulation networks enabling the targeted adaptation through the sensing of a wide range of environmental conditions.

## Tuning Biofilms Architecture to Control Their Functions?

**FIGURE 3:**
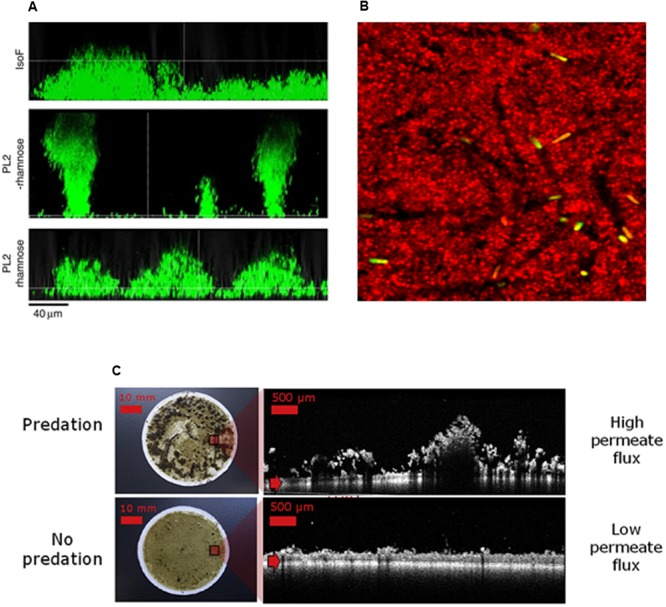
Sculpting biofilm architecture to control their function. **(A)** Role of biosurfactants in *Pseudomonas putida* biofilm architecture. Images displayed vertical sections of biofilms grew in flow chamber during 3 days in presence or absence of rhamnoses. IsoF correspond to the wild-type strain and PL2 strain to a conditional mutant in which the native promoter region of psoA (a gene coding a large non-ribosomal peptide synthethase which directs the biosynthesis of the two cyclic lipopeptide biosurfactants putisolvin I and II) has been replaced with the rhamnose-inducible PrhaB promoter (adapted from Carcamo-Oyarce et al., 2015). Addition of 0.2% rhamnose in growth medium of PL2 lead to the recovery of the flat wild-type biofilm structure suggesting that putisolvins promote the colonization of the substratum. **(B)** Predation by protozoa affects biofilms spatial organization during gravity-driven dead-end ultrafiltration and induces higher permeate fluxes (adapted from Derlon et al., 2012). **(C)** Green bacilli creates transient pores in the biofilm matrix of *Staphylococcus aureus*, leading to an increased sensitivity to biocide action as described in Houry et al. (2012) (courtesy of Julien Deschamps, INRA).

Sculpting biofilm spatial organization represents an attractive approach to control their overall functions, whether beneficial or deleterious (**Table 1**). The structure of those surface-associated communities can be faceted by governing their local environmental or by exposing them to molecular and biological effectors. Illustrations of such shaping are displayed in **Figure 3**.

### Manipulating Biofilm Local Environment

Within a biofilm, individual cells have the ability to monitor their direct environment (nutrients, pH, ionic strength, oxygen, surface…). The integration of these various external signals leads to specific cellular responses that can be exploited to alter the community structure/function.

In this line, Sauer et al. (2004) elegantly demonstrated that a sudden increase in carbon substrate or pH of the growing medium lead to significant change in *P. aeruginosa* biofilm structure. Changing the glutamate concentration of the media from 2 to 20 mM triggers a total loss of the biofilm tridimensional structure in less than 60 min. This massive loss of surface-associated biomass observed was correlated with the induction of a subpopulation of bacteria with an increased expression of flagella and a decreased expression of pilus, allowing their dispersal in the flow. Similarly, *Staphylococcus epidermidis* biofilm exposed to a high osmotic pressure (from 86 to 776 mM NaCl) decreased the average bacterial local number density by 10-fold (Stewart et al., 2013). Increasing the flow shear stress applied on *P. aeruginosa* biofilm reduced the formation of self-aggregating clusters, in particular through a significant down regulation of genes involved in extracellular polysaccharide synthesis (Crabbé et al., 2008; Dingemans et al., 2016). Exposing a gravity sewer biofilm to increasing shear stress (from 1.12 to 1.45 mPa) affected porosity of the biostructure (from 70 down to 55%) and reduced the chemical oxygen demand in the sewers from 40 to 32% (Xu et al., 2017). Growing the microaerophilic human pathogen *Campylobacter jejuni* under aerobic condition (20% O_2_) stimulates the kinetic of biofilm development (Reuter et al., 2010) and the complexity in their architecture (Turonova et al., 2015). Desiccation of the biofilm occurs periodically in various environments including soils, industrial surfaces or hypersaline ponds (Habimana et al., 2014; Decho, 2016; Lennon and Lehmkuhl, 2016). In the latter environment, the EPS attains a glass state upon extreme desiccation that presumably protects the biofilm inhabitants and allows them to resume activities upon rehydratation. When grown at the air interface, *Bacillus subtilis* developed a biofilm protected by a hydrophobic raincoat layer formed by the BslA surface-active protein (Arnaouteli et al., 2016). This interfacial layer of water-repellent proteins also protects the biofilm inhabitants from ethanol and biocide action (Epstein et al., 2011). When the biofilm structure limits antimicrobial penetration and prevents the contact with the microbial target, exposition to pulsating waves of energy (e.g., ultrasonic waves) can amplify the antimicrobial effect. This so called bio-acoustic effect is likely associate with a deformation of the biofilm and a better penetration in the EPS of the toxic compounds (Qian et al., 1996; Peterson et al., 2015). Another often neglected environmental parameter to shape biofilm is the nature of the substratum. Greene et al. (2016) demonstrated that it was possible to alter the biofilm structure of *Acinetobacter baumannii* only by changing its carrier nature. While an important structured biofilm was able to grow in 4 days on polycarbonate coupons, only sparse adhering cells were visible in the same condition on glass (biofilm biovolume decreased from more than 2.5 to below 0.1 μm^3^/μm^2^). Not only the spatial arrangement of the cell were altered by the nature of the substratum, but also the bacterial physiology as reported by the live/dead ratio that ranged from less than 2 for biofilm grown on rubber to almost 8 for cell grown on stainless steel. Muszanska et al. (2012) demonstrated that coating silicone rubber with a brush polymer alters the biofilm structure (including a strong decrease in the polysaccharidic matrix) and the susceptibility to the gentamycin antibiotic. From those observations, authors suggested that the antimicrobial treatments of biofilm-associated infections could be more effective on material protected with such active antibiofilm coatings. Similarly, Valle et al. (2006) observed that treating abiotic surfaces with group II capsular polysaccharides drastically reduces both initial adhesion and biofilm architecture by important nosocomial pathogens.

All these examples illustrate the possibility to manipulate the structure/function association of microbial biofilms by controlling one (or a combination) of parameter(s) in their local environment.

### Reprogramming Biofilm Structure/Function with Specific Molecular Triggers

External cues can be put in used to act both directly on the biofilm EPS properties or reprogram individual cell physiology and transcriptional expression patterns. A large palette of exogeneous enzymes has the ability to degrade specific moieties of the complex biofilm matrix. Those EPS-degrading enzymes can act specifically on extracellular polysaccharides (dispersin B), proteins (proteinase K, trypsin) or eDNA (DNase I) (Boles and Horswill, 2011). Cocktails of such enzymes are proposed in the food-industry to target persistent deleterious biofilms (Lequette et al., 2010; Nguyen and Burrows, 2014). Dispersin B that hydrolyzes the glycosidic linkages of PNAG was found to be efficient in a range of pathogenic bacteria and is being commercially developed as a wound care gel (Kaplan et al., 2003). Enzymes from bacteriophages can dissolve extracellular polysaccharides of the matrix and reverse the biofilm tolerance to antibiotics and other antimicrobial treatments (Chan and Abedon, 2015). Bacteriophage enzymes were able to reduce the alginate EPS viscosity by up to 40% in *P. aeruginosa* biofilm (Hanlon et al., 2001). Using purified EPS depolymerase isolated from an *Enterobacter agglomerans* bacteriophage, Skillman et al. (1998) demonstrated a change in the physical properties of the EPS from a two species biofilms resulting in the effective removal of both species. Another telling example is the use of the biosynthetic glycoside hydrolases PelAh and PslGh that were able to disrupt the spatial organization of a pre-existing *P. aeruginosa* biofilm within 1 h, potentiating the action of colistin and neutrophils (Baker et al., 2016). By targeting the cell wall, the hydrolases LySMP was able to reduce the biofilm structure of *Streptococcus suis* by more than 80% and facilitate the action of several antibiotics on sessile communities (Meng et al., 2011).

Amyloids fibers are the “neglected child” of the EPS matrix (Dueholm and Nielsen, 2016) while evidence is rinsing that those proteinous assemblages are important drivers of the matrix viscoelastic properties (Lembré et al., 2014). D-amino acids (with some controversy) and parthenolide were identified as molecular inhibitors targeting the polymerisation or anchorage to the cell wall of TasA, the main *Bacillus subtilis* EPS amyloid (Kolodkin-Gal et al., 2010; Leiman et al., 2013; Romero et al., 2013). *P. aeruginosa* produces *cis*-2-decenoic acid, a small messenger molecule responsible for the induction of the biofilm dispersion response in a range of Gram-negative and Gram-positive bacteria. It has been shown to alter biofilm structure and to reverse tolerance to conventional antimicrobial agents (Marques et al., 2015). The matrix reprogrammation can also be triggered by biofilm cells exposition to sublethal concentration of antimicrobials. Schilcher et al. (2016) observed that subinhibitory concentrations of clindamycin upregulated the expression of major biofilm-associated genes in *S. aureus* biofilm and shift the composition of the biofilm matrix toward higher eDNA content.

In addition to soluble molecular effectors, microorganisms are able to respond to organic and inorganic volatiles in their local headspace, some of which influencing their ability to form biofilm (Audrain et al., 2015). Nitric oxide (NO) is a volatile messenger able to trigger biofilm dispersion. Barraud et al. (2009) demonstrated that exposing a multispecies biofilms in water system to 500 nM sodium nitroprusside (NO donor) almost totally abolished the biofilm spatial organization, increasing by 20 the efficacy of the conventional chlorine treatment. On the opposite, ammonia, a volatile produced by many bacteria, stimulates biofilm formation in *Bacillus licheniformis* and other relatives (Nijland and Burgess, 2010). Similarly, it was showed that self-produced acetic acid was used as volatile signals to stimulate and coordinate the timing of biofilm formation in *B. subtilis* (Chen et al., 2015). This behavioral biofilm response triggered by odorant molecules was compared to olfaction; it opens doors to new biofilm control strategies based on airborne volatile metabolites.

### Guided Biofilm Ecology to Shape the Biofilm Structures and Functions

As mentioned previously, biofilm architecture and functions are intimately related to their microbial content and the spatial repartition of their inhabitants. In, several fields including health, agriculture, food processing and environment, new strategies emerged to manipulate biofilm functions by guided biofilm ecology. The effectors of these approaches are selected organisms that can alter population structures in the targeted community such as bacteria, bacteriophages, molds, yeasts, microalgae, amoeba, and metazoans.

A family of microbial probiotics are put in used on the market to combat human biofilm-associated infections (Vuotto et al., 2014). Specific inhabitants of the oral microbiome such as *Porphyromonas gingivalis* are responsible of the production of unpleasant malodorant volatile sulfur compounds (halitosis) (Lee and Baek, 2014). Different reports described a beneficial long term effect of combining conventional oral mouthwashes chemical pretreatment with probiotic therapies involving lactic acid bacteria such as the bacteriocin producing *Streptococcus salivarius* K12 (Masdea et al., 2012; Jamali et al., 2016). Using an agent-based spatially explicit model approach, Bucci et al. (2011) demonstrated that the competitive dynamic of bacteriocin producing strain in a multispecies biofilm strongly depends on a single critical bacteriocin-range parameter that measures the threshold distance from a focal bacteriocin-producing cell whose fitness is higher than that of sensitive cell. Similarly, the biofilm of *Aggregatibacter actinomycetemcomitans* involved in chronic periodontal diseases was degraded after exposition to a Lactobacillus probiotic altering the biofilm structure (Jaffar et al., 2016). *Lactobacillus rhamnosus* GG and *Lactococcus lactis* HY449 both affect the spatial organization of model oral biofilms and reduced the count of oral pathogens in the community (Jiang et al., 2016; Kim and Lee, 2016). The most widespread use of probiotic is the treatment of gastrointestinal diseases. Rieu et al. (2014) demonstrated that a Lactobacillus-induced host immunomodulation response was strongly enhanced when the potential probiotic was cultivated as a structured biofilm in contrast with free-cells. This fundamental discovery leads to the exploration of new biofilm-based formulations to increase their *in vivo* beneficial effects (Cheow et al., 2014).

In the medical area, an emerging research field to overcome bacterial antibioresistance (super bugs) and chronic biofilm-associated infections (BAI) is the bacteriophage therapy (Maura and Debarbieux, 2011; Chan and Abedon, 2015). In the lab, bacteriophages were efficient in mice models to treat a (biofilm associated) *P. aeruginosa* acute lung infection (Debarbieux et al., 2010). Exposing *Clostridium difficile* colony biofilms to a cocktail of selected phages lead to the emergence of lysed zones and elongated cells morphotypes in the structure, but the loss of cell viability observed in early stages decreased with biofilm age (Nale et al., 2016). While highly effective on free-cells, the architecture of biofilm, the diversity of cell types and the presence of matrix likely limit the phages efficacy to treat chronic BAI. Only few human phase II trials explored this approach to treat human patient with only mitigated success (Wright et al., 2009). There is a clear need for larger scale trials and deeper research on phage and biofilm interactions in this promising emerging field (Servick, 2016).

From farms to forks, the microbiological control of raw and processed food through the food chain is still mainly ensure by the use of chemical products including pesticides, antibiotics or disinfectants. Their massive use raised some important environmental and health concerns and stressed out the need for alternative sustainable approaches. In the crop field, a recent paper pinpointed the biofilm mode of life as an important driver of the efficacy of microbial biocontrol agents (Pandin et al., 2017). Indeed, different studies showed evidence that biocontrol agent are able to form protective biofilms on crop that develop antagonistic properties against unwanted microorganisms (Zeriouh et al., 2014). The associated mechanisms likely involved many of the biofilm traits, including spatial competition, cell-cell signaling and the production of antimicrobials (Zhou et al., 2016). Spraying antagonistic *Bacillus subtilis* TSK1-1 or *Bacillus amyloliquefaciens* WG6-14 on citrus leaf surface alter the spatial organization and the density of *Xanthomonas axonopodis* pv. *citri*, a pathogenic bacteria involved in citrus canker (Huang et al., 2012). A comparative transcriptome analysis of the biocontrol agent *Bacillus amyloliquefaciens* FZB42 as response to biofilm formation showed an up regulation of the *lci* gene encoding an antimicrobial peptide, and of operons involved in the production of the extracellular matrix (Kröber et al., 2016). It was also shown that the architecture of those protective biofilm can be stimulated by plant metabolites such as root exudates (Espinosa-Urgel et al., 2002). Similar protecting biofilms are envisioned in the feed/food environments to protect livestock building and the surface of food processing equipments from pathogen persistence (Mariani et al., 2011; Piard and Briandet, 2016). Habimana et al. (2011) demonstrated using confocal imaging and a simplified individual based model that exposing sessile cells of *Listeria monocytogenes* to *Lactococcus lactis* engaged a spatial race to interfacial nutrients resulting in a total loss of the pathogen multiplication. It was also recently shown that motile bacilli can create transient pores in *Staphylococcus aureus* biofilms, sensitizing the pathogenic structure to biocide action (Houry et al., 2012).

The use of organisms to shape new biofilm functions is also emerging in environmental sciences. Derlon and his collaborators nicely demonstrated that predation mediated by added protozoa (*Tetrahymena pyriformis*) triggers strong architectural change of an ultrafiltration membrane biofilm (from flat to heterogeneous and porous structure), increasing by 2 the permeate flux (Derlon et al., 2012). The same group also demonstrated that metazoan worms, including the nematode *Plectus aequalis* and the oligochaetes *Aelosoma hemprichi*, were also able to remodel membrane fouling biofilm structure and to increase significantly the membrane efficacy (Derlon et al., 2013; Klein et al., 2016).

The microflora of stone monuments is mainly composed of microbial biofilms and lichens. Scientists of this field implicate these complex ecosystems in stone damage while others pinpointed their bioprotective role (Pinna, 2014). Application of biofilm-induced calcium carbonate precipitation is an emerging tool for the bioremineralisation of stone and cultural heritage (Dhami et al., 2014). Dick et al. (2006) evaluated the performance of *Bacillus sphaericus* biofilms to restore deteriorated Euville limestone, a stone used for building and sculpturing in France. They demonstrated an important surface colonization and the presence of dense calcium carbonate crystals on biofilms formed on the treated stone. Similar biocalcifying effect was observed with *Bacillus subtilis* on deteriorated globigerina limestone (Micallef et al., 2016).

Environmental biofilms are largely involved in global biogeochemical cycles (Singer et al., 2010). Through human intensive activities and the resulting environmental changes, we are unintentionally affecting and remodeling those natural ecosystems. At the Paris climate conference (COP21) in 2015, 195 countries adopted a legally binding global climate deal. The agreement sets out a global action plan to put the world on track to avoid dangerous climate change by limiting global warming to well below 2°C above pre-industrial levels (Rhodes, 2016). Indeed, a 2°C warming in flowing water is already enough to drive significant changes in freshwater biofilm structure/function by inducing a complex reorganization in the network of interactions among microbial populations within the biofilm matrix (Romani et al., 2014).

## Conclusion

Architectural plasticity of biofilm constitutes a central process to actively adapt to stress and to increase productivity and fitness of microbial communities in response to changing environmental conditions. Considering dynamics of biofilm structure is thus required to better understand the emergence of novel functional properties and to decipher the communal mechanisms underlying microbial behavior, from single cell to multicellular community. Although our ability to predict and manage the functional properties and adaptation strategies of these complex dynamic communities is yet limited, the increasing development of predictive modeling approaches and the improvement of integration of experiments and models should, in a near future, enable to better link composition, dynamic organization and function of microbial communities (Widder et al., 2016). Recent technological advances in single-cell analytic methods have led to the generation of quantities of novel interesting data on individual microbial behaviors which still are to be exploited through individual-based modeling approach for instance, to provide insights into self-organized spatial patterns and to construct a realistic vision of biofilm at both the individual and community levels (Hellweger et al., 2016).

## Author Contributions

All authors listed have made a substantial, direct and intellectual contribution to the work, and approved it for publication.

## Conflict of Interest Statement

The authors declare that the research was conducted in the absence of any commercial or financial relationships that could be construed as a potential conflict of interest.
